# Supplementary material for: Accurate prediction of functional states of cis-regulatory modules reveals common epigenetic rules in humans and mice
Source: BMC Biol. 2022 Oct 5;20:221. doi: 10.1186/s12915-022-01426-9 (PMC9535988; doi:10.1186/s12915-022-01426-9)
Supplement: Supplementary file 2 — Additional file 2: Figure S1. Number of positive (active) CRMs predicted by dePCRM2 in a cell/tissue type. Figure S2. Distributions of phyloP scores of predicted CRM candidates and non-CRMs in the human (A) and mouse (B) genomes. Figure S3. Boxplots of MACS signal scores of the four epigenetic marks on pooled positive and negative sets defined by the seven methods (Table 1), as well as on pooled two negative sets CRM+S- and non-CRM, in the six human cell lines. Figure S4. Distributions of phyloP scores of pooled positive sets and negative sets from the six human cell lines, defined by the seven methods (Table 1) in comparison with that of the covered 85.5% human genome regions. Figure S5. Heatmaps of signals of the four epigenetic marks around positive sets CRM+TF+ and CRM+S+, and negative sets non-CRM and CRM+S- in the A549, HepG2, K562 and MCH-7 cells. Figure S6. ROC curves of the LR models trained on the 15 combinations of the four marks in the 67 human cell/tissue types. Figure S7. Using additional epigenetic marks does not significantly improve prediction accuracy in the human cell/tissue types. Figure S8. Identification of optimal minimal sets of epigenetic marks for predicting functional states of CRMs in 64 mouse cell/tissue types. Figure S9. ROC curves for the 15 combinations of the four marks in the mouse cell/tissue types. Figure S10. Adding more epigenetic marks to the four does not significantly improve prediction accuracy of functional states of CRMs in mouse cell/tissue types. Figure S11. Distribution of the distances of the putative CRMs to their nearest transcription start sites (TSSs) in the human (A) and mouse (B) genomes. Figure S12. Performance of the models for differentiating active proximal CRMs and active distal CRMs. Figure S13. Genome-wide predictions of active CRMs in a mouse cell/tissue type and their reutilizations in different cell/tissue types. Figure S14. Predicted uniquely active CRMs in the human and mouse cell/tissue types. [file 12915_2022_1426_MOESM2_ESM.docx]

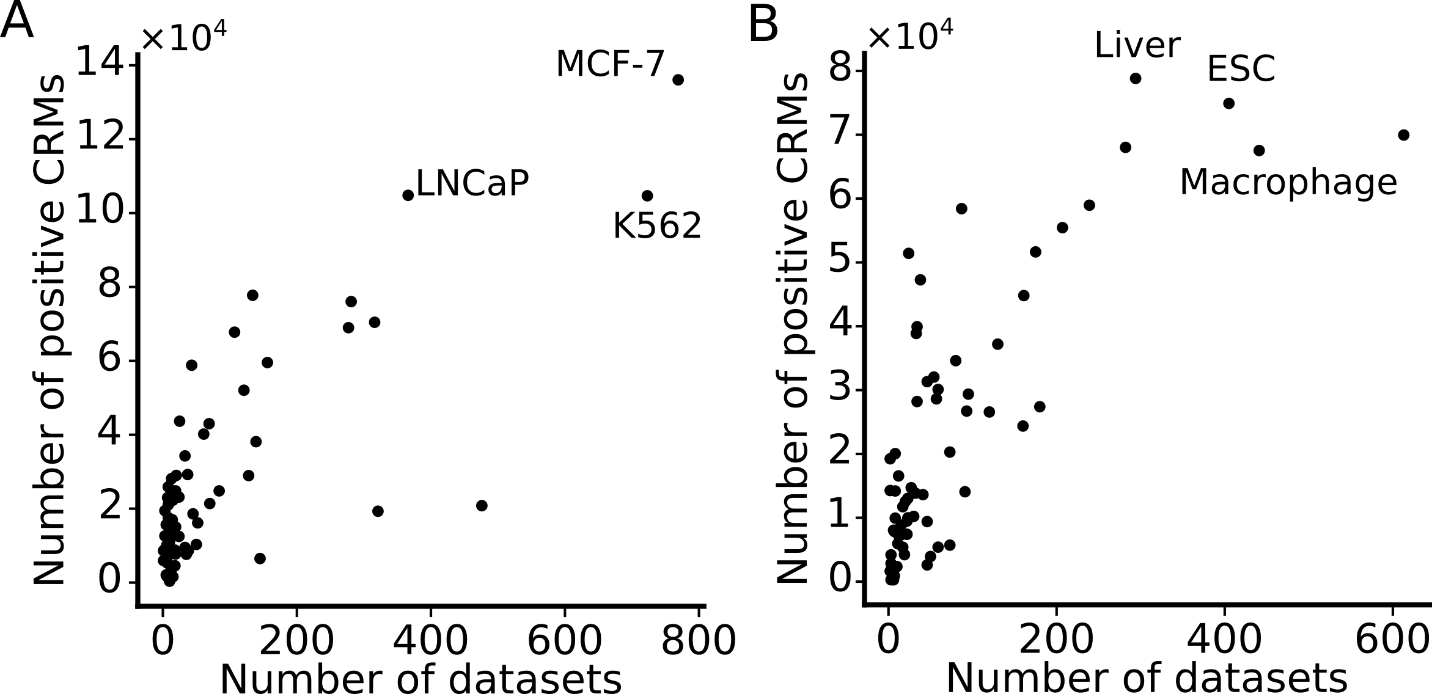


**Fig. S1. Number of positive (active) CRMs predicted by dePCRM2 in a cell/tissue type.** Number of predicted active CRMs in the 67 human (**A**) or 64 mouse (**B**) cell/tissue types as a function of the number of TF ChIP-seq datasets available in the cell/tissue types. Cell/tissue types with a larger number of predicted active CRMs are indicated. These varying numbers of predicted active CRMs in each cell/tissue type are used as the positive sets (CRM+TF^+^) to train machine-learning classifiers.

**
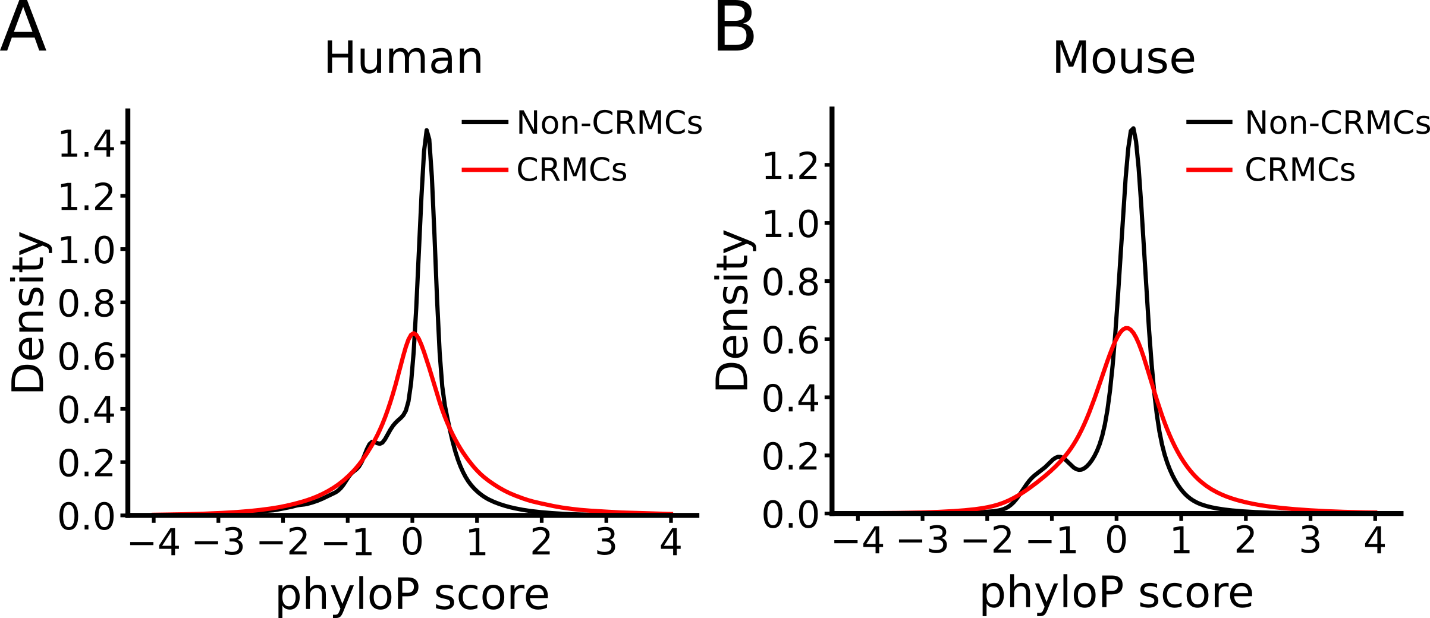
**

**Fig. S2. Distributions of phyloP scores of predicted CRM candidates and non-CRMs in the human (A) and mouse (B) genomes.** The predicted CRM candidates in both genomes are more likely under either strongly positive selection (with negative phyloP scores) or strongly negative selection (with positive phyloP scores), while the predicted non-CRMs are largely selectively neutral (with near zero phyloP scores).


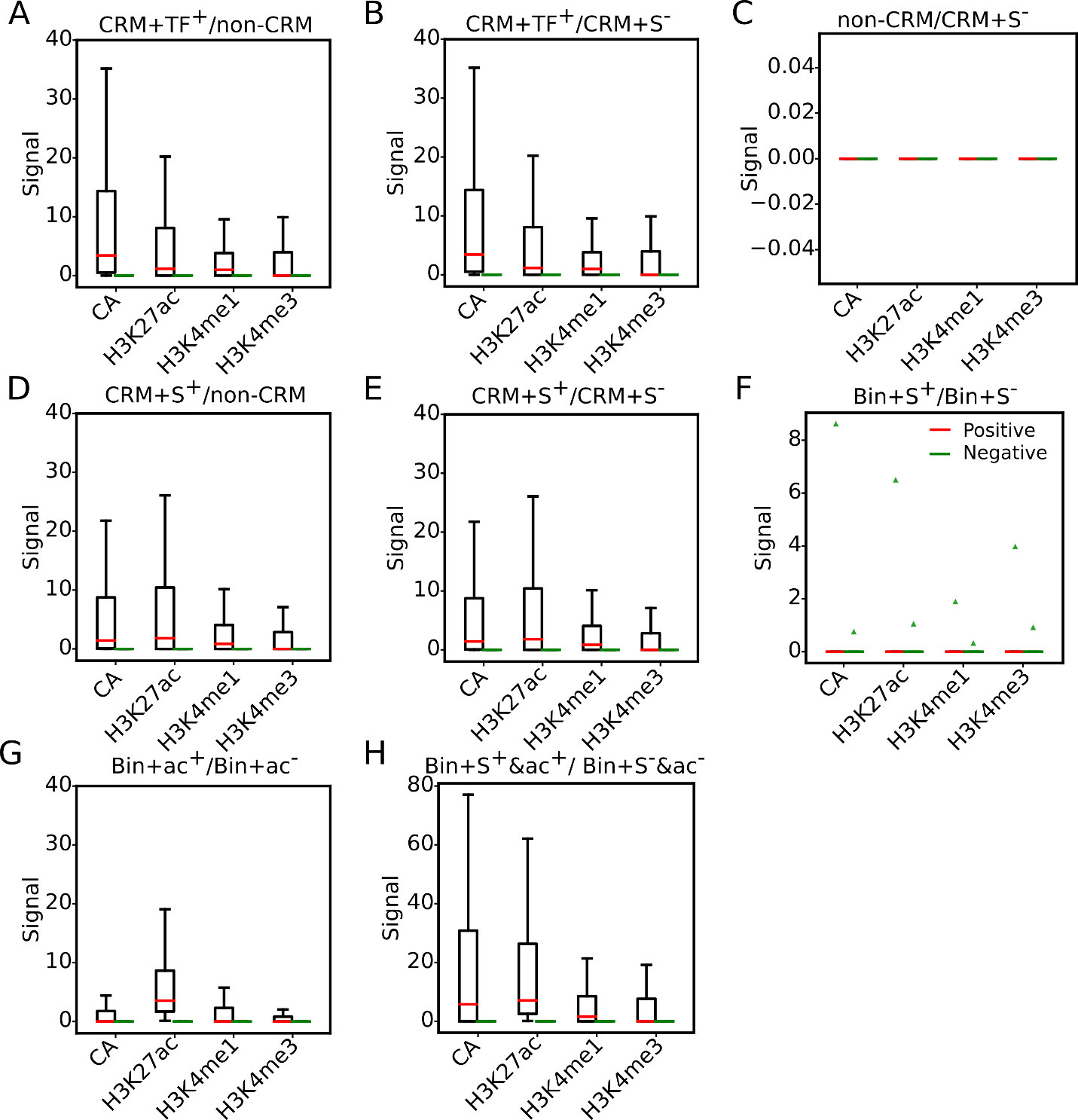


**Fig. S3. Boxplots of MACS signal scores of the four epigenetic marks on pooled positive and negative sets defined by the seven methods (Table 1), as well as on pooled two negative sets CRM+S^-^ and non-CRM, in the six human cell lines.** **A.** CRM+TF^+^/non-CRM. **B.** CRM+TF^+^/CRM+S^-^. **C.** non-CRM**/**CRM+S^-^. **D.** CRM+S^+^/non-CRM. **E.** CRM+S^+^/CRM+S^-^. **F.** Bin+S^+^/Bin+S^+^. **G**. Bin+ac^+^/Bin+ac^-^. **H.** Bin+S^+^&ac^+^/Bin+S^-^&ac^-^.


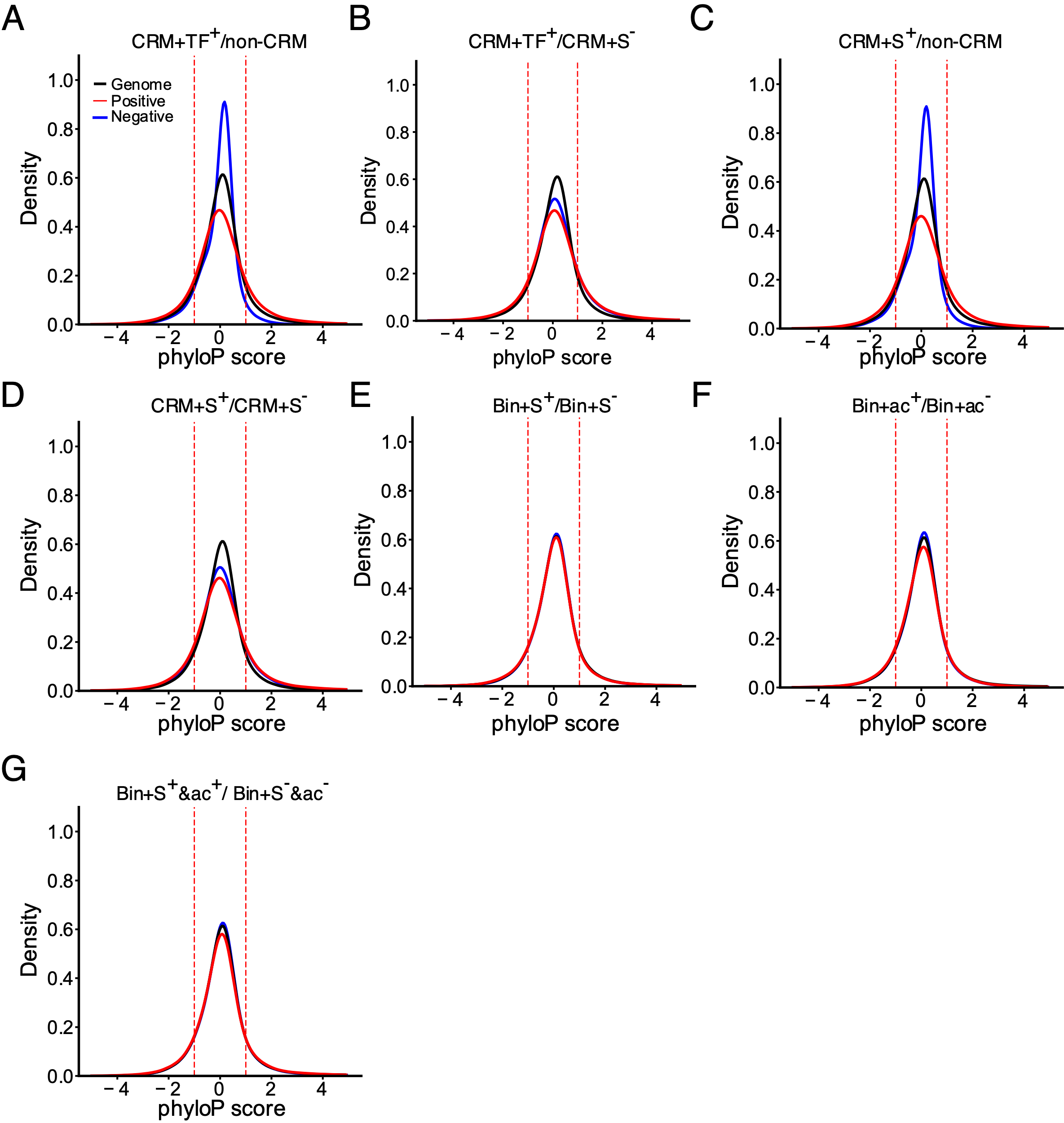


**Fig. S4. Distributions of phyloP scores of pooled positive sets and negative sets from the six human cell lines, defined by the seven methods (Table 1) in comparison with that of the covered 85.5% human genome regions. A.** CRM+TF^+^/non-CRM. **B.** CRM+TF^+^/CRM+S^-^. **C.** CRM+S^+^/non-CRM. **D.** CRM+S^+^/CRM+S^-^. **E.** Bin+S^+^/Bin+S^-^. **F**. Bin+ac^+^/Bin+ac^-^. **G.** Bin+S^+^&ac^+^/Bin+S^-^&ac^-^.


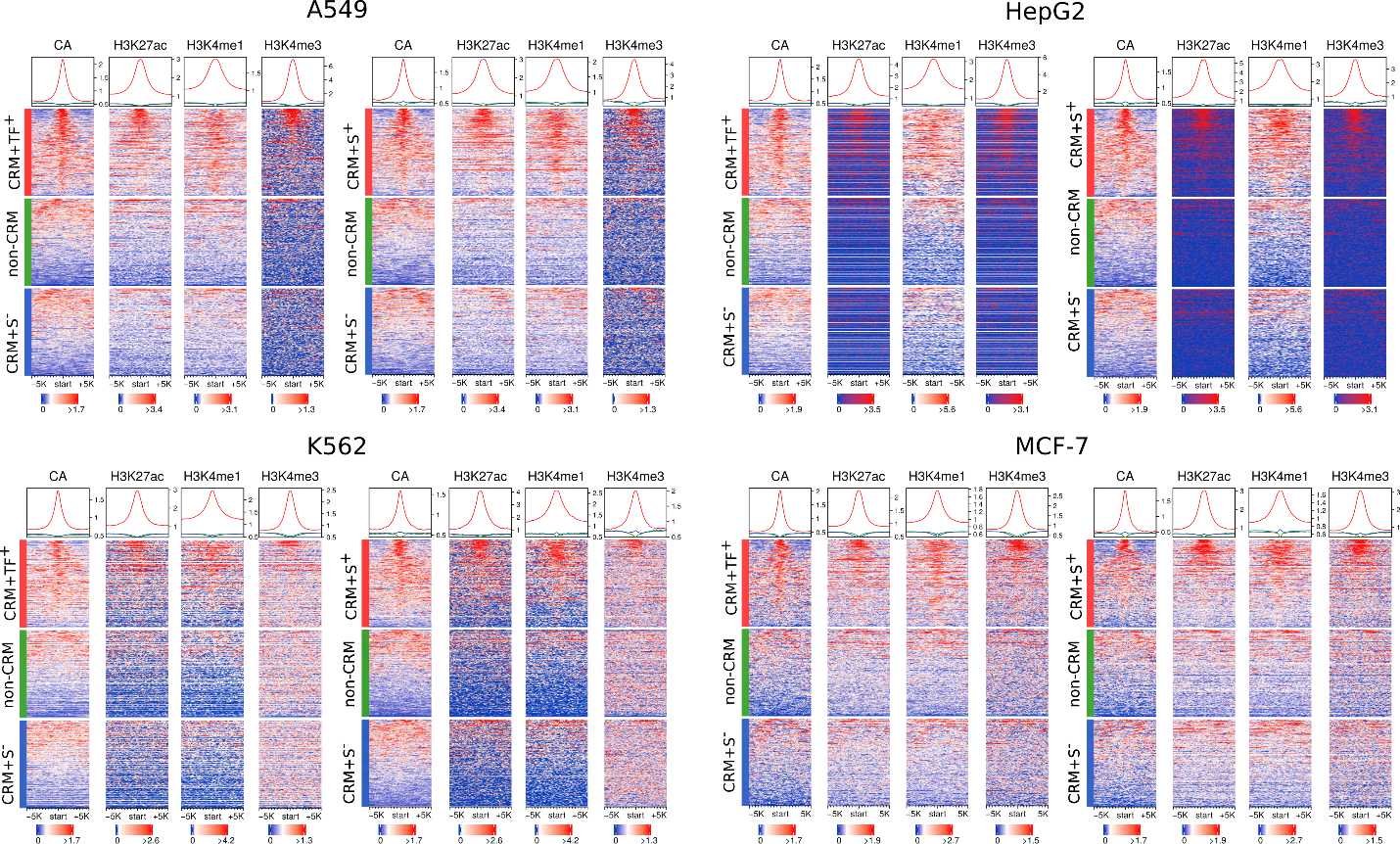


**Fig. S5. Heatmaps of signals of the four epigenetic marks around positive sets CRM+TF^+^ and CRM+S^+^, and negative sets non-CRM and CRM+S^-^ in the A549, HepG2, K562 and MCH-7 cells.** We plotted signals of CA, H3K4me1, H3K3me3, and H3K27ac around the sequences in each set. To make the density plots, we first extended the centers of sequences to 10 kb, and then for each 100-bp tiling window in the extended regions, we calculated the signal scores using EnrichedHeatmap (w0 mode) (Gu et al. 2018). Note that the scales of vertical axes of the density plots on the top of the heatmaps are different in the right and left panels for each cell line. The sequences in each set are sorted in the descending order of the CA signals for all the marks.


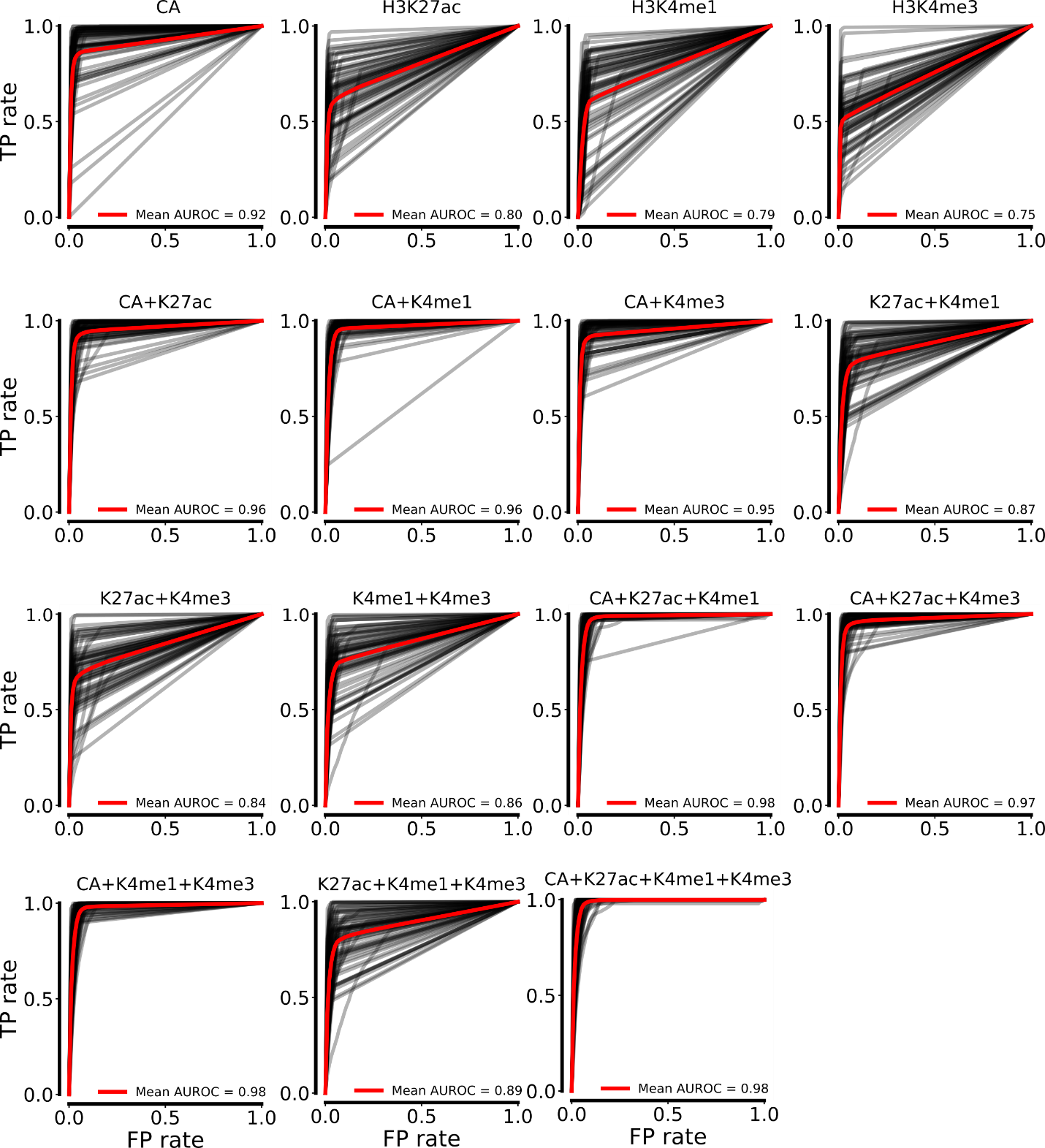


**Fig. S6. ROC curves of the LR models trained on the 15 combinations of the four marks in the 67 human cell/tissue types.** Each gray curve is the ROC curve for a cell/tissue type, and the red one is the mean of ROC curve for the 67 human cell/tissue types.


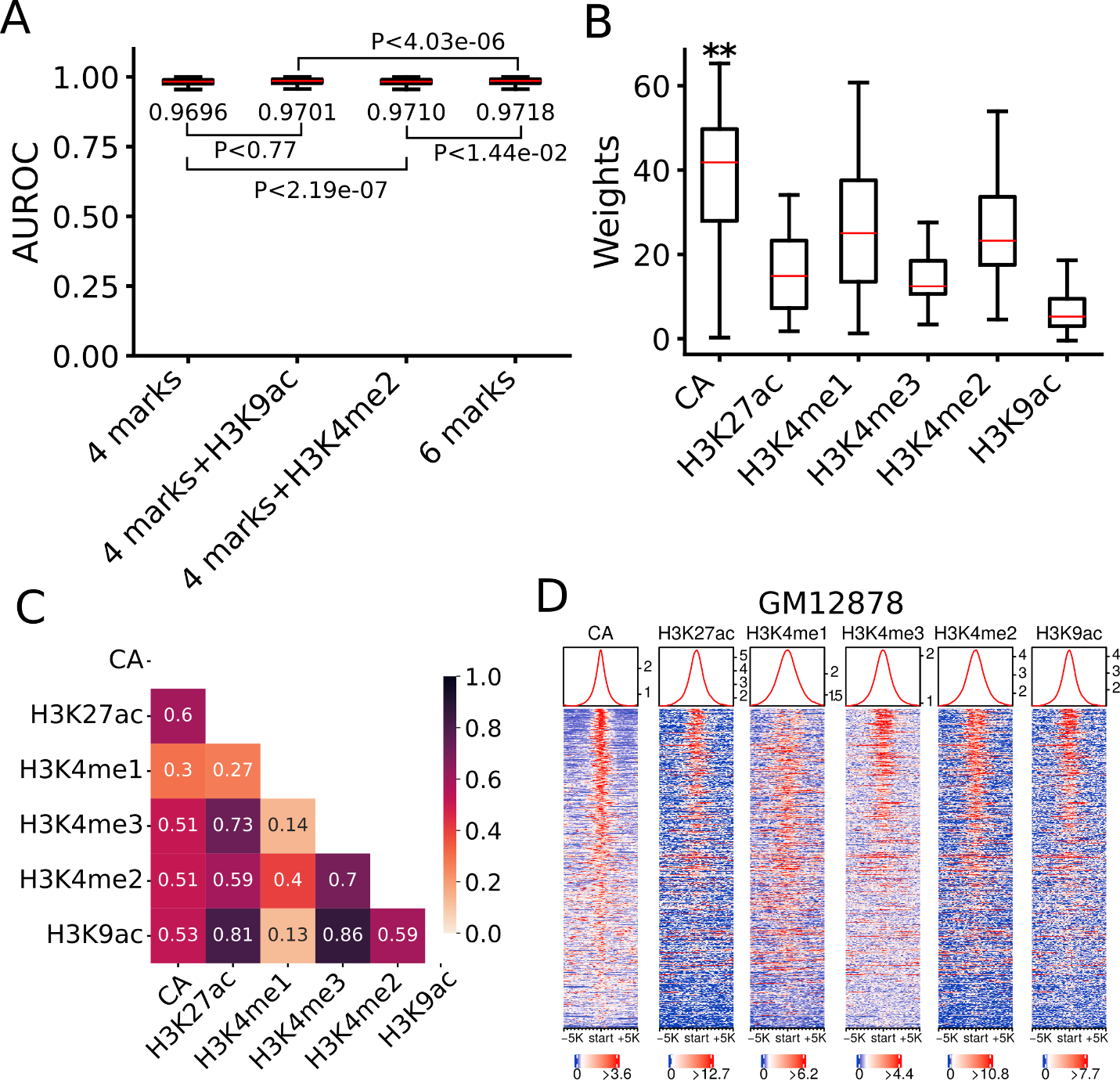


**Fig. S7. Using additional epigenetic marks does not significantly improve prediction accuracy in the human cell/tissue types**. **A.** Boxplot of AUROCs of the LR models trained and tested in each of the 22 human cell/tissue types (10-fold cross-validation) using the four (CA, H3K4me1, H3K4me3 and H3K27ac), five (adding H3K4me2 or H3K9ac) and six (adding H3K4me2 and H3K9ac) marks**.** The statistical tests were done using Wilcoxon signed-rank test. **B.** Boxplot of coefficients of the six marks in the LR models trained on the 22 cell/tissue types using the six marks. **p<0.001 (Wilcoxon signed-rank test), the weights of CA are significantly higher than those of the other three marks. **C.** Mean Pearson’s correlation coefficients between signals of different marks in the 22 cell/tissue types. **D.** Heat map of the signals of the six epigenetic marks around the positive set CRM+TF^+^ in the GM12878 cells, showing correlations between signals of different marks.


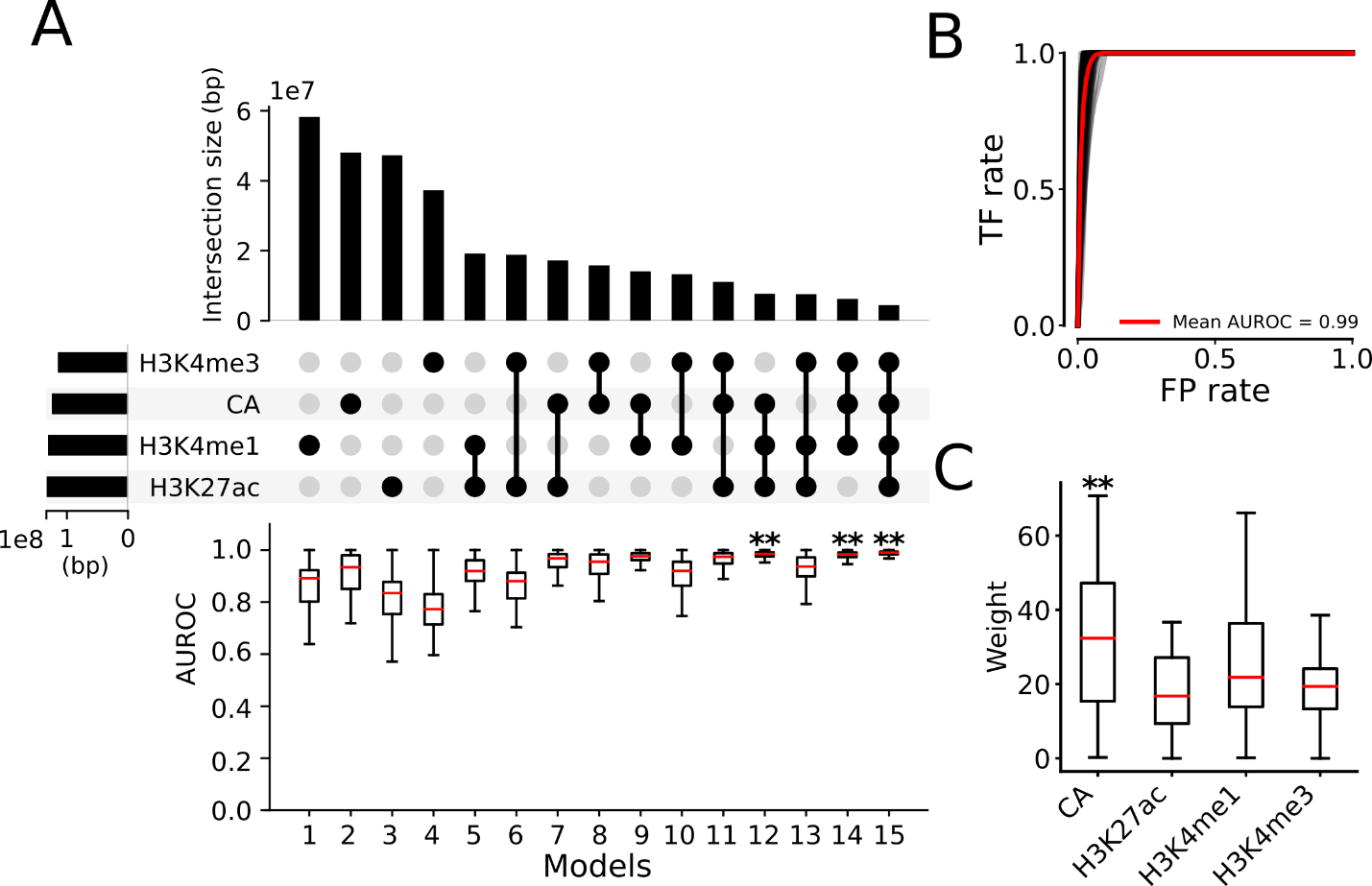


**Fig. S8. Identification of optimal minimal sets of epigenetic marks for predicting functional states of CRMs in 64 mouse cell/tissue types. A.** Upset plot showing the mean unique genome positions of the peaks of each mark and the mean intersections of genome positions covered by the peaks of the four epigenetic marks (upper and middle panels), and boxplot of AUROCs of the 15 LR models using the 15 different combinations of the four marks in the 64 mouse cell/tissue types. Models 12, 14 and 15 have similar AUROCs, each is significantly higher than those of the other 12 models; **p<0.001 (Wilcoxon signed-rank test). **B.** ROC curves of model 15 in the 64 mouse cell/tissue types. Each gray curve is the ROC curve for a cell/tissue type, and the red one is the mean ROC curve of the 64 cell/tissue types. **C.** Boxplot of weights (coefficients) of the four marks in model 15. **p<0.001 (Wilcoxon signed-rank test), the weights of CA are significantly higher than those of the other three marks;


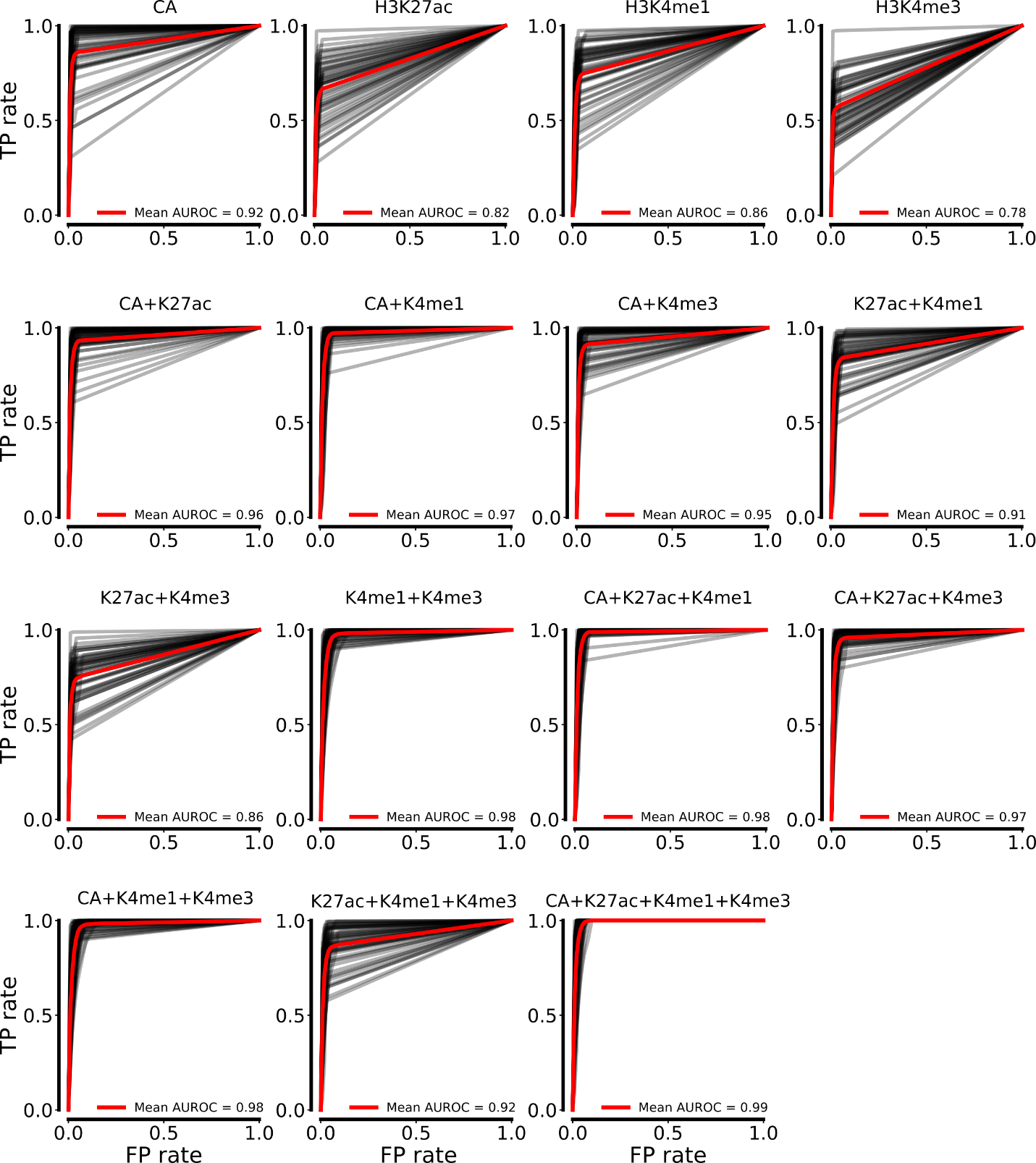


**Fig. S9. ROC curves for the 15 combinations of the four marks in the mouse cell/tissue types.** Each gray curve is the ROC curve for a cell/tissue type, and the red one is the mean of ROC curve for the 64 mouse cell/tissue types.


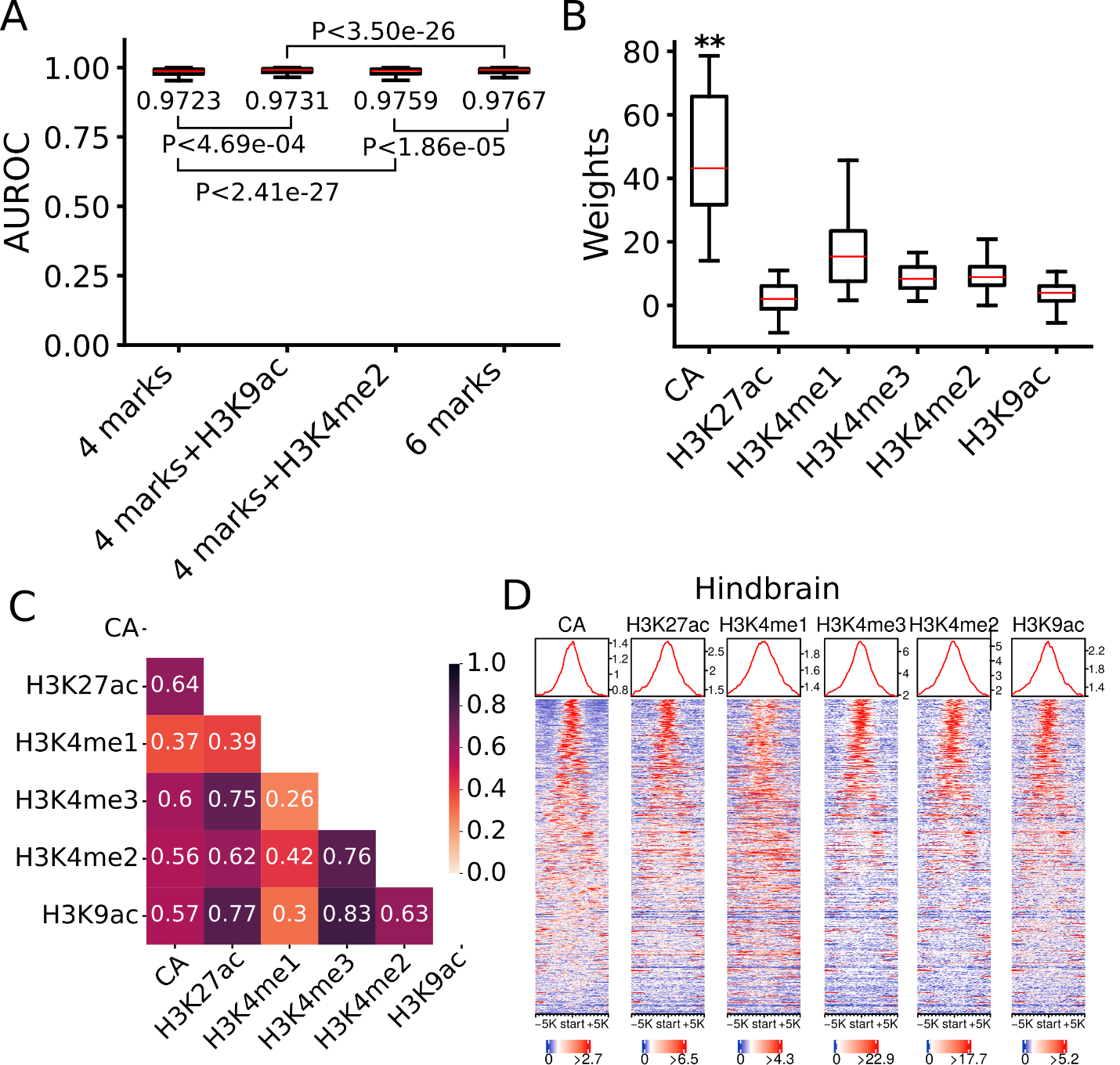


**Fig. S10. Adding more epigenetic marks to the four does not significantly improve prediction accuracy of functional states of CRMs in mouse cell/tissue types. A.** Boxplot of AUROCs of models trained and tested on each cell/tissue type (10-fold cross-validation) using the four(CA, H3K4me1, H3K4me3 and H3K27ac), five (adding H3K4me2 or H3K9ac) and six (adding H3K4me2 and H3K9ac) marks. The statistical tests were done using Wilcoxon signed-rank test. **B.** Boxplot of coefficients of the six marks in the LR models trained on the 29 cell/tissue types using the six marks. **p<0.001 (Wilcoxon signed-rank test), the weights of CA are significantly higher than those of the other three marks. **C.** Mean Pearson’s correlation coefficients between signals of different marks in the 29 human cell/tissue types. **D.** Heat maps of the signals of the six epigenetic marks around positive set CRM+TF^+^ in the mouse hindbrain cells, showing correlations between signals of different marks.


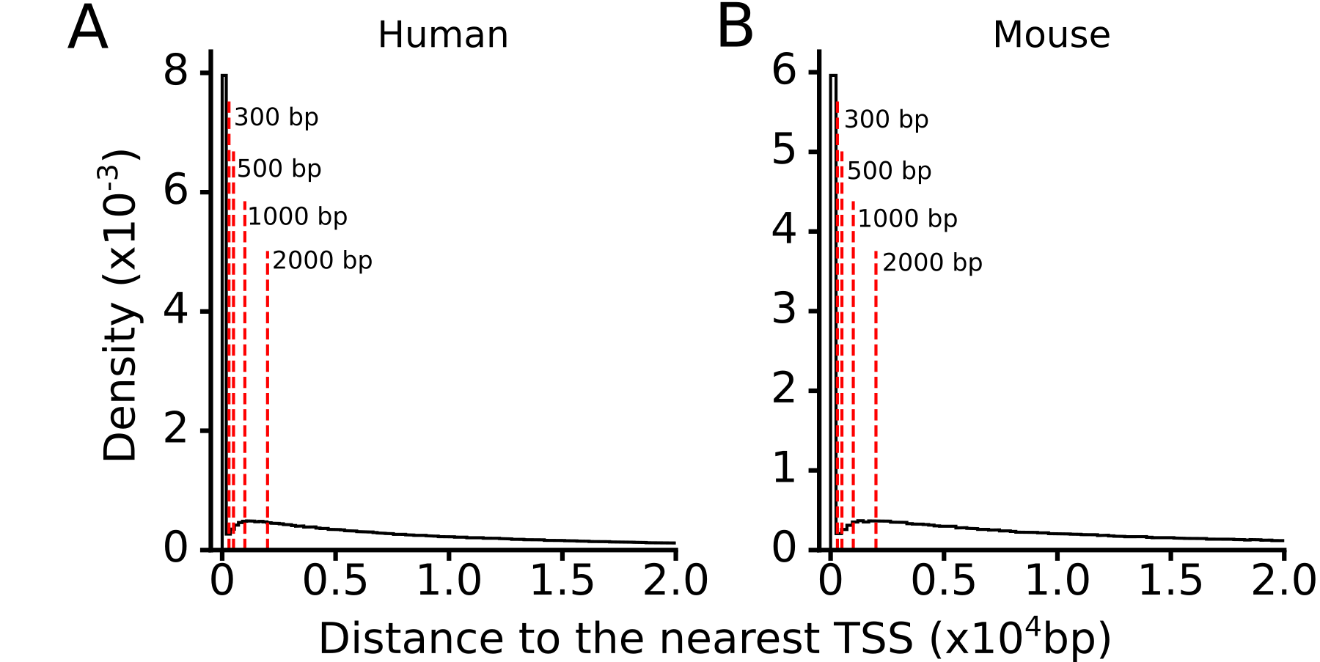


**Fig. S11.** **Distribution of the distances of the putative CRMs to their nearest transcription start sites (TSSs) in the human (A) and mouse (B) genomes.**


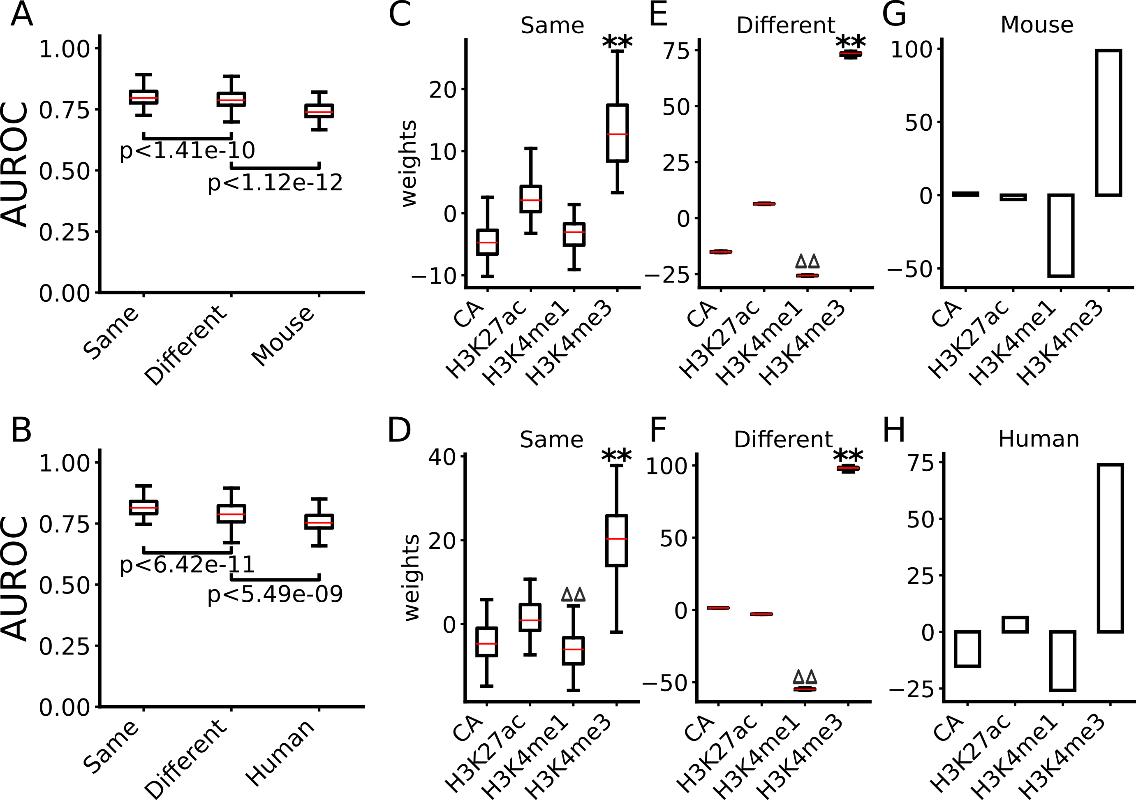


**Fig. S12. Performance of the models for differentiating active proximal CRMs and active distal CRMs. A, B.** Boxplots of AUROCs of the models trained and tested in the same cell/tissue types in human (A) or mouse (B), of the models trained in multiple cell/tissue types in human (A) or mouse (B) and tested in different cell/tissue types in the same species, and of the model trained in multiple cell/tissue types in mouse (A) or human (B) and tested in various cell/tissue types in human or mouse, respectively. The statistical tests were done using Wilcoxon signed-rank test. **C, D.** Boxplots of coefficients of the four marks in the models trained and tested in the same cell/tissue types in human (C) or mouse (D). **E, F.** Boxplots of coefficients of the four marks in the models trained in multiple cell/tissue types in human (E) or in mouse (F) and tested in different cell/tissue types in the same species. **p<0.001 (Wilcoxon signed-rank test), the weights of the labeled mark are significantly higher than those of the other three marks; ^∆∆^p<0.001 (Wilcoxon signed-rank test), the weights of the labeled mark are significantly lower than those of the other three marks. **G, H.** Coefficients of the four marks in the model trained in multiple cell/tissue types in mouse (G) or human (H) and tested in various cell/tissue types in human, or mouse, respectively.


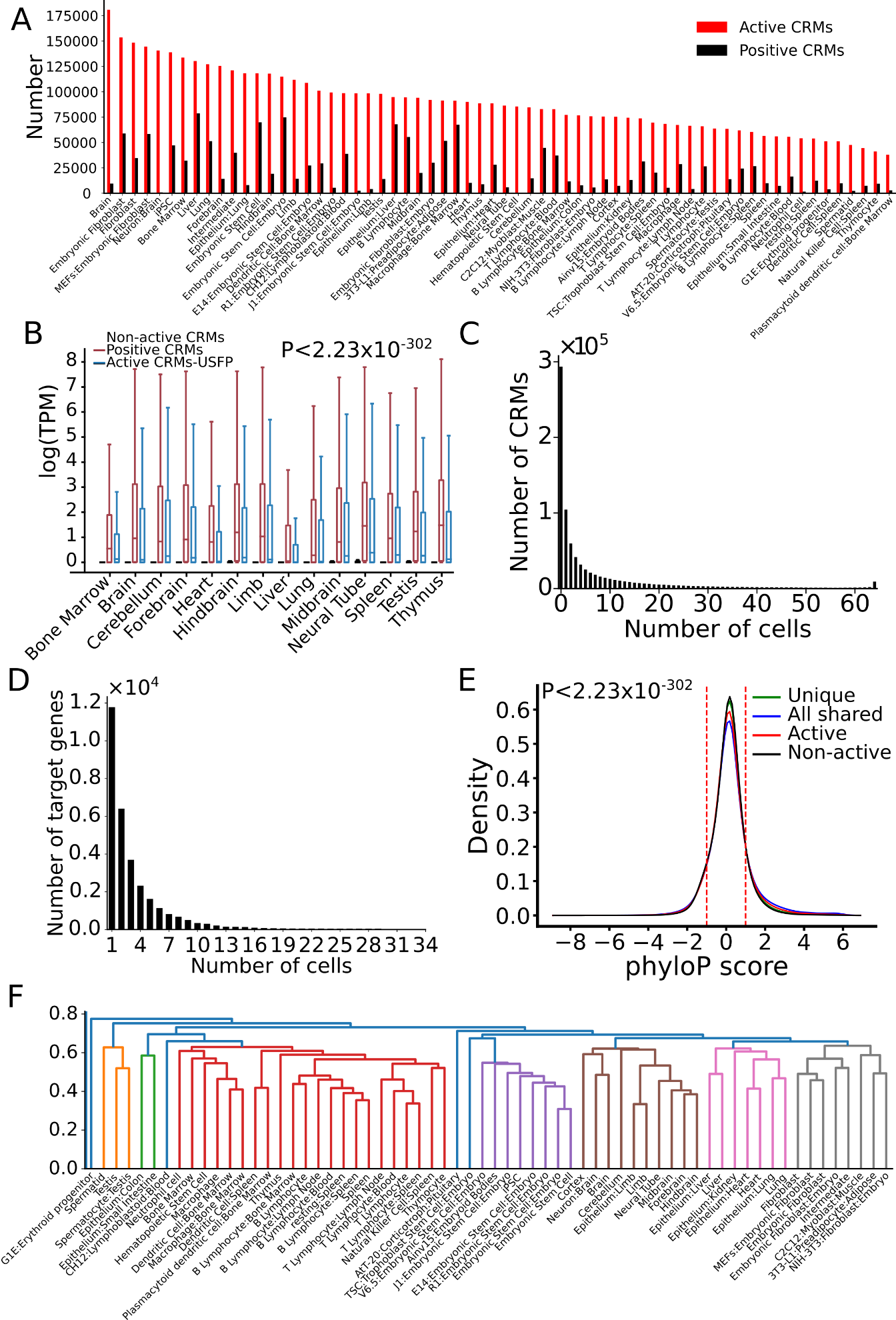


**Fig. S13. Genome-wide predictions of active CRMs in a mouse cell/tissue type and their reutilizations in different cell/tissue types. A.** Number of active CRMs predicted by the UFSP (active CRMs) versus the size of the positive set CRM+TF+ (positive CRMs) in a cell/tissue type. **B.** Boxplots of gene expression levels in a cell/tissue type showing that genes closest to the CRM+TF^+^ set (positive) or to the active CRMs predicted by the UFSP model but missed by dePCRM2 (active CRMs-UFSP) have significantly higher expression levels than genes closest to the predicted non-active CRMs (p<2.23X10^-302^, Mann Whitney U test). **C.** Number of predicted active CRMs shared by different numbers of cell/tissue types. **D.** Number of closest genes to the uniquely active CRMs shared by different numbers of cell/tissue types. **E.** Distributions of phyloP scores of all-shared active CRMs, uniquely active CRMs, all active CRMs and all non-active CRMs in the cell/tissue types. All distributions are significantly different from one another, p<2.23X10^-302^ (K-S test). **F.** The levels of shared active CRMs reflect lineage relationships of the cell/tissue types. Cell/tissue types were clustered based on the Jaccard index of predicted active CRMs in each pair of the cell/tissue types.


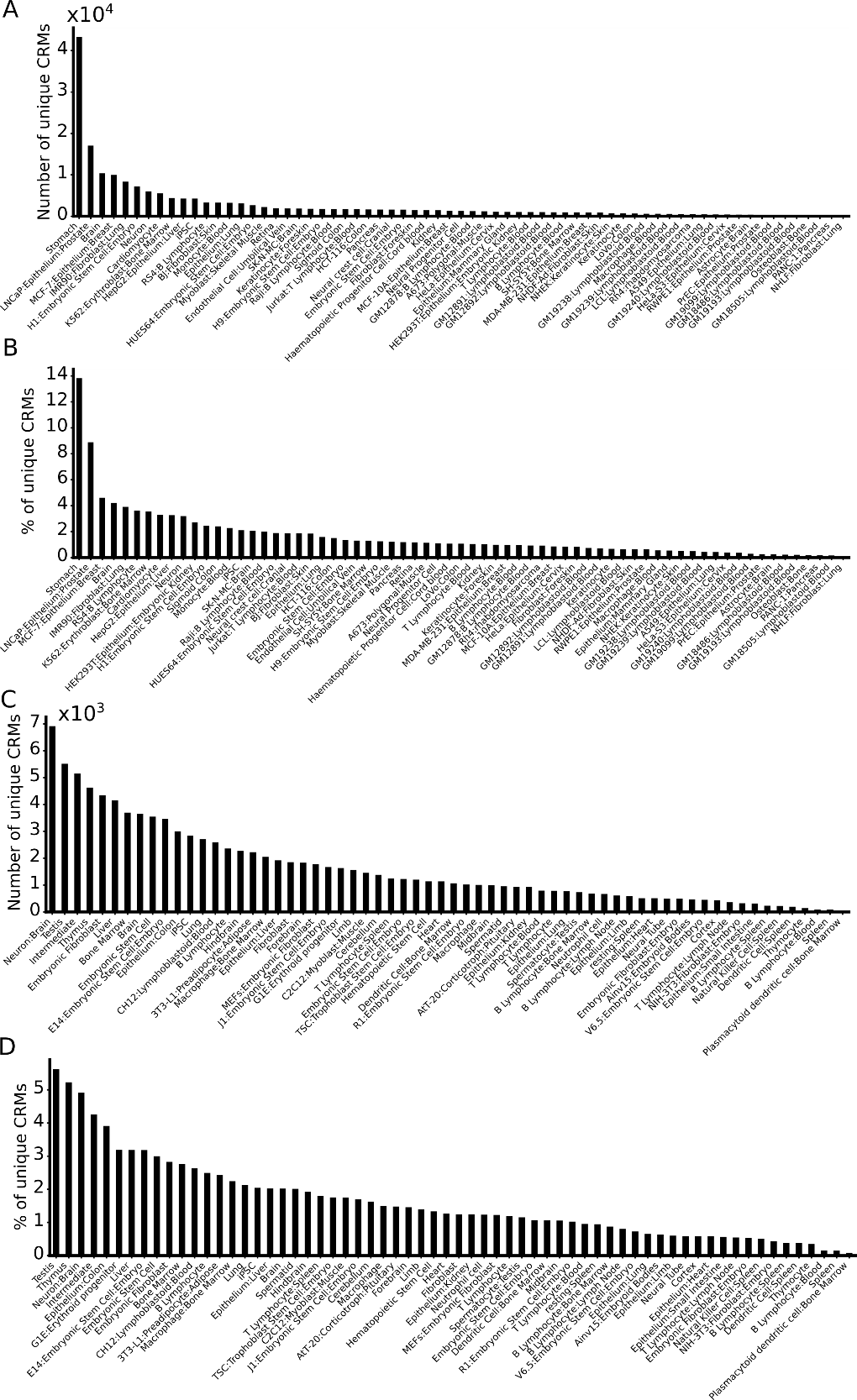


**Fig. S14. Predicted uniquely active CRMs in the human and mouse cell/tissue types**. **A, C.** Number of predicted uniquely active CRMs in each of the human (A) and mouse (C) cell/tissue types. **B, D.** Percentage of predicted uniquely active CRMs in each of the human (B) and mouse (D) cell/tissue types.
